# Regulatory role of S1P and its receptors in sepsis-induced liver injury

**DOI:** 10.3389/fimmu.2025.1489015

**Published:** 2025-01-28

**Authors:** Bin Wang, Xiaoyu Wu, Jiangfeng Cheng, Junming Ye, Hongquan Zhu, Xiaofeng Liu

**Affiliations:** ^1^ The First Clinical College, Gannan Medical University, Ganzhou, Jiangxi, China; ^2^ Department of Critical Care Medicine, The First Affiliated Hospital of Gannan Medical University, Ganzhou, Jiangxi, China; ^3^ Clinical College, Suzhou Medical College of Soochow University, Suzhou, Jiangsu, China; ^4^ Department of Critical Care Medicine, The First Affiliated hospital of Gannan Medical University, Ganzhou, Jiangxi, China; ^5^ Department of Emergency, The First Affiliated Hospital of Gannan Medical University, Ganzhou, Jiangxi, China

**Keywords:** S1P, S1PRs, sepsis, immune, bile acid metabolism, liver-intestinal circulation

## Abstract

As an immune and metabolic organ, the liver affects the progression and prognosis of sepsis. Despite the severe adverse effects of sepsis liver injury on the body, treatment options remain limited. Sphingosine-1-phosphate (S1P) is a widely distributed lipid signaling molecule that binds to five sphingosine-1-phosphate receptors (S1PR) to regulate downstream signaling pathways involved in the pathophysiological processes of sepsis, including endothelial permeability, cytokine release, and vascular tone. This review summarizes current research on the role of S1P in normal liver biology and describes the mechanisms by which changes in S1P/S1PR affect the development of liver-related diseases. At the same time, the pathological processes underlying liver injury, as evidenced by clinical manifestations during sepsis, were comprehensively reviewed. This paper focused on the mechanistic pathways through which S1P and its receptors modulate immunity, bile acid metabolism, and liver-intestinal circulation in septic liver injury. Finally, the relationships between S1P and its receptors with liver inflammation and metabolism and the use of related drugs for the treatment of liver injury were examined. By elucidating the role of S1P and its receptor in the pathogenesis of sepsis liver injury, this review established a molecular targeting framework, providing novel insights into clinical and drug development.

## 1 Introduction

Sepsis is a life-threatening condition hallmarked by organ dysfunction caused by a dysregulated host response to infection (1, 2). A 2016 systematic review estimated the current epidemiology of sepsis at approximately 30 million exacerbations, resulting in 6 million deaths annually worldwide (3). Owing to its special anatomical location, the liver is one of the most critically affected organs by sepsis (4). According to epidemiological studies, the average incidence of liver dysfunction in patients with sepsis is 39.9%, with the mortality rate of septic patients with liver insufficiency or failure as high as 54%-68% (5). Notably, a significant relationship was identified between the occurrence or deterioration of liver dysfunction and mortality during septic shock (6). The pathogenesis of sepsis-induced liver injury is intricate and is associated with an imbalance between pro-inflammatory and anti-inflammatory responses caused by the activation of inflammatory factors, the generation of oxygen free radicals and subsequent peroxide reaction, the obstruction of bile acid secretion and excretion, the vicious circle involving the gut-liver axis, hypoperfusion-induced microcirculation disturbances, hepatocyte autophagy, direct or indirect hepatocyte apoptosis caused by endotoxins, and so on (7, 8). Thus, liver dysfunction can severely compromise the outcome of sepsis and has been identified as a strong independent predictor of mortality in the intensive care unit (9).

Sphingosine-1-phosphate (S1P) is a signaling lipid that is ubiquitous in human body fluids, tissues, and cells (10). S1P biosynthesis occurs in the endoplasmic reticulum, where L-serine and palmitoyl-CoA undergo a series of reduction, transformation, and modification reactions to form ceramide, which is deacylated into sphingosine and finally phosphorylated by two sphingosine kinases, namely SphK1 and SphK2, to form S1P (11, 12). The cytosolic level of S1P is regulated by sphingosine-1-phosphate phosphatase and sphingosine-1-phosphate lyase (13). Specifically, S1P can be dephosphorylated by the former or irreversibly degraded to phosphoethanolamine and hexadecenal by the latter (13). S1P is predominantly distributed in the serum and lymph in the human body and is primarily transported by concentration gradients (14). Moreover, it is exported from cells and can act as an autocrine and paracrine signaling molecule in the surrounding environment or in distant tissues (15).

S1P can regulate various pathophysiological processes in sepsis, including endothelial permeability, cytokine release, and vascular tone (16). Furthermore, it exerts a significant influence on the physiological and pathological changes in the immune system, lung tissue, and liver tissue by binding to different G protein-coupled receptors (GPCRS), namely S1PR1-5 (17, 18). S1P and its receptors can further alleviate hepatocyte injury and liver metabolic disorders by mitigating the inflammatory response and improving vascular permeability in sepsis (19, 20). In clinical trials, serum S1P concentration was lower in patients with sepsis compared to normal controls, with the loss of serum S1P being correlated with disease severity (21, 22). The distribution and action of S1P can affect the degree of liver injury in sepsis (23). For instance, in mouse models of lipopolysaccharide (LPS)-induced liver injury and inflammation, LPS-induced inflammation up-regulates hepatic S1PR1 and S1PR3 expression (24, 25). Pretreatment of mice with 4-deoxypyridoxine (DOP) or the sphingosine analog FTY720 attenuated the disruption in pulmonary and hepatic vascular barriers after the development of sepsis (26). While existing evidence indicates that S1P and its receptors may play a key role in mediating immunity and metabolism in sepsis-induced liver injury, a systematic review of the literature is lacking. Therefore, this article reviewed the recent research progress on S1P and its receptors as potential therapeutic targets in the immunoregulation and metabolism of sepsis-induced liver injury.

## 2 Distribution and expression of S1P and S1PRs in liver physiological metabolism and immune cells

As is well documented, the liver is a vital organ that regulates S1P levels in the blood (27, 28). Indeed, hepatectomy has been demonstrated to reduce plasma S1P levels, suggesting that the liver may be involved in regulating plasma S1P levels *in vivo (* 29).

Extracellular vesicles (EVs) are small lipid bilayer-enclosed nanovesicles released by various cell types and play a key role in intercellular communication, including the transmission of inflammatory signals, promotion of angiogenesis and tissue remodeling (30). The EV membrane is rich in cholesterol and sphingomyelin (31). In lipotoxicity-induced liver inflammation, EVs secreted by hepatocytes are rich in S1P and mediate the chemotaxis of macrophages through the S1P1 receptor (32). Studies have shown that hepatocyte-derived exosomes may be potential biomarkers of liver disease (33). In addition, exosome-induced hepatocyte proliferation depends on the intracellular production of S1P (33).

S1P is released from primary synthetic cells, and during transportation, approximately 60% of S1P is bound to apolipoprotein M (ApoM) in high-density lipoprotein (HDL), whilst the remaining 40% is bound to albumin (34). However, ApoM is typically synthesized in the liver (35). Plasma S1P levels were reduced by 50% in apoM-deficient mice, demonstrating a direct correlation between circulating S1P levels and ApoM (36). The same connection exists in humans (37). In the liver, S1PR1-5 receptor subtypes are present (38, 39). Among them, S1PR1, S1PR2, and S1PR3 are all highly expressed in the liver. In contrast, the levels of S1PR4 and S1PR5 expression are too low to be detectable (40).

S1P is closely related to inflammatory markers (41). In circulation, S1P bound to HDL exerts an effective anti-inflammatory effect on smooth muscle cells by inhibiting the expression of inflammatory genes stimulated by TNF-α (42). However, surprisingly, S1P exhibits completely opposite effects when binding to different receptors. The SPHK1/S1P/S1PR1 signaling pathway plays a crucial role in sphingolipid metabolism and signal transduction (25). Studies have shown that the activation of SPHK1 further triggers downstream molecules such as TRAF-2, NFκB, Ras, and PLC, promoting the production of inflammatory factors and affecting cell survival, proliferation, migration, and apoptosis (25). Additionally, S1P promotes the generation of TNF-α, IL-1, IL-6, and monocyte chemoattractant proteins through S1PR2, and S1PR2 can also activate the RhoA/ROCK signaling pathway, leading to pyroptosis of THP-1 cells (43, 44). Moreover, in macrophages, S1P is catalyzed by SphK1 and binds to S1PR3 to regulate aerobic glycolysis and influence macrophage polarization, mediating the inflammatory response in sepsis (45). S1PR4 expression is downregulated in LPS/IFN-γ-induced primary M1 macrophages, but no significant changes are observed in M2 macrophages (46). Subsequent studies have shown that the deficiency of S1PR4 promotes the activation of pro-inflammatory macrophages (46). In the research of chronic obstructive pulmonary disease, it was found that the increased expression of S1PR5 may lead to macrophage dysfunction (47).

S1P plays a decisive role in liver physiology and metabolism (48). S1PR2 can be activated by taurocholate (TCA) and other conjugated bile acids, which in turn regulates hepatic lipid and sterol metabolism by up-regulating the expression and activity of SphK2, increasing the gene expression of nuclear S1P, inhibiting specific histone deacetylases (HDAC), and increasing histone acetylation (49, 50). In addition to the promoting role shown in lipid metabolism, S1P also plays a pivotal role in governing glucose metabolism, glycogen deposition, and lipid accumulation in hepatocytes (51). For example, ApoM/S1P exerts protective effects against insulin resistance by activating insulin signaling pathways, such as AKT and AMPK pathways, to enhance mitochondrial function (52). Additionally, bile acid-activated S1PR2 displays insulin-like activity in hepatic glucose regulation, whereas S1PR2 antagonists prevent palmitic acid-mediated insulin resistance (53). Therefore, the determination of S1P levels in bile and its target organs, such as the liver, bile ducts, and intestine, is essential to further elucidate the role of S1P in the pathophysiology of hepatobiliary diseases (54).

The liver is considered to be one of the major organs of the immune system and is principally subdivided into parenchymal and nonparenchymal cells (55). Hepatic parenchymal cells are hepatocytes (HCS) and cholangiocytes, which account for 60% to 80% of liver tissue and function as part of the “hepatic immune system” (56). Nonparenchymal cells, namely liver sinusoidal endothelial cells (LSECs), liver satellite cells, Kupffer cells, neutrophils, monocytes, T and B lymphocytes, natural killer (NK) cells, and NKT cells, also possess immune functions (57). Collectively, these cells constitute liver physiological functions, including nutrient metabolism, drug detoxification, and immune response (58). Of note, immune cells make up a large proportion of the liver composition (59). For example, macrophages are abundant in healthy rodent livers, with 20 to 40 macrophages per 100 hepatocytes (55). A diverse range of S1PR expression patterns was noted in the immune system (**Table 1**).

**Table 1:** S1P receptors and their distribution and effects on immune cells.

Cell types	Receptor	Effect	Reference
Macrophages	S1PR1	It regulates the polarization of macrophages	(59, 156)
	S1PR2	It drives NLRP3 inflammasome activation and secretion of inflammatory cytokines (interleukin-1β and interleukin-18) in BMMs	(100)
	S1PR3	It down-regulates the expression of NLRP3 and pro-IL-1β during LPS priming	(101)
Neutrophils	S1PR2	It stimulates neutrophil infiltration in bile duct ligation-induced liver injury in mice	(69)
DC cells	S1PR1	It regulates dendritic cell trafficking	(157)
	S1PR2	–	
	S1PR3	It regulates migration and endocytosis of mature DCS	(158)
	S1PR4	Plasmacytoid dendritic cell differentiation	(159)
	S1PR5	–	
NK cells	S1PR5	As a chemokine receptor, it mediates the distribution of NK cells in the body and their trafficking to the lesion site	(160)
T cells	S1PR1	It inhibits T cell proliferation, cytokine (IFN-γ and IL-4) production, and migration and enhances naive T cell survival	(107, 161–163)
	S1PR4	It inhibits the proliferation of T cells and the secretion of effector cytokines (IL-2, IFN-γ, and IL-4) and concomitantly enhances the secretion of inhibitory cytokine IL-10	(162, 164)
B cells	S1PR1	It inhibits B cell migration and inflammatory factors	(165, 166)
	S1PR2	It maintains germinal center B cell homeostasis	(167)
	S1PR3	It promotes normal B cell development	(168)

The S1PR expression patterns detailed in this table reflect those known to be expressed at detectable levels on the cell surface.

## 3 Role of S1P and its receptors in liver diseases

Recent studies have reported that the S1P/S1PR axis also plays an integral role in acute liver failure, metabolic syndrome, non-alcoholic fatty liver disease, liver fibrosis, and liver cancer (60). For example, S1P plays a key role in acetaminophen-induced acute liver injury by promoting endoplasmic reticulum stress and mitochondrial permeability transition (61) and also affects cancer progression by regulating histone deacetylase (HDAC1/2) and NF-κB pathways (62). The S1P/S1PR1 axis delays the development of alcoholic liver disease by preventing the migration of Th17 (helper T cells) and their accumulation in the liver and intestine (63). Meanwhile, SphKs and its product S1P have been described to regulate hepatocyte apoptosis and survival (39). In summary, sphingosine-1-phosphate (S1P) regulates pathophysiological processes in the liver, encompassing liver regeneration, endothelial barrier, and immune response (64).

### 3.1 Liver fibrosis

In a previous study, S1P levels measured by HPLC were elevated in human fibrotic livers, accompanied by an increased expression level of S1PR1 and S1PR3 in myofibroblasts (65, 66). This may be ascribed to the upregulation of S1PR1 and S1PR3, facilitating the differentiation of bone marrow mesenchymal stem cells (BMSC) into myofibroblasts (67). S1PR1/3 antagonists can attenuate the degree of liver fibrosis by suppressing the up-regulation of angiogenesis markers in the damaged liver (65). In addition, S1PR2-siRNA can alleviate liver fibrosis and inflammation by inhibiting the formation of neutrophil extracellular traps (NET) in fatty liver model mice (68). During the early stages of chronic liver disease, S1PR2-mediated activation of neutrophils is implicated in the progression of liver injury and may potentially serve as a novel therapeutic target for cholestatic liver disease (69). Inflammation driven by the NLRP3 inflammasome is a key trigger of liver fibrosis during cholestatic liver injury (70). S1P promotes the activation of the NLRP3 inflammasome and the secretion of pro-inflammatory cytokines (IL-1β and IL-18) through the S1PR2/Gα pathway (71). Treatment with JTE-013, an S1PR2 inhibitor, significantly limited the initiation and activation of the NLRP3 inflammasome in cholestatic liver injury (40, 71). In conclusion, different S1P/S1PR pathways exert distinct effects at various stages of liver fibrosis.

### 3.2 Nonalcoholic steatohepatitis

The development of Nonalcoholic steatohepatitis (NASH) is linked to endoplasmic reticulum (ER) stress and necessitates the co-localization of Caspase-2 with S1P (72). Thus, the interrelationship between lipid accumulation, cytokines, and lysophospholipids plays a central role in the pathogenesis of NASH (72). In a mouse model of NASH, liver S1P levels and Sphk1 levels were increased (73). Reem Rida et al. pointed out using HepG2 cells that FTY720P binds to S1PR3 and sequentially activates Gq and phosphatidylinositol 3-kinase (PI3K), which can promote lipid accumulation (73). In an experimental NASH animal model, S1PR1 knockdown inhibited saturated fat-induced NF-κB signaling and decreased the mRNA expression level of TNFα and monocyte chemoattractant protein 1 (MCP1) in HepG2 cells (74). The role of S1P in steatohepatitis is highlighted by the fact that the administration of S1P antagonists to experimental NASH animals eradicates the disease (75). Taken together, these data establish a mechanistic link between sphingolipid signaling and hepatic lipid accumulation and inflammation in NASH, providing valuable insights for the treatment of this disease.

### 3.3 Hepatocellular carcinoma

S1P has been shown to promote cell proliferation, migration, invasion, and epithelial-mesenchymal transition (EMT) in Hepatocellular carcinoma (HCC) (60). In an *in vitro* experiment, the HCC cell line HepG2 was found to secrete S1P, thereby preventing cell proliferation (76). FTY720 not only exerts potent antiangiogenic effects but is also cytotoxic to cancer cells and has been found to effectively inhibit the proliferation of numerous cancer cells (77). At present, FTY720 is regarded as a novel immunosuppressant with potent anticancer properties (78) In the MCA-RH7777 hepatocellular carcinoma cell line model, FTY720 reduced intrahepatic and pulmonary metastases as well as tumorigenesis, possibly through its function as an S1PR1 modulator (79). FTY720 also induced apoptosis in HCC cells by activating protein kinase Cδ signaling (80). In addition, FTY720 inhibited the invasion and proliferation of HCC by down-regulating S1PR1 expression and inhibited the recurrence of HCC after liver transplantation (81). Lastly, Fty720 significantly prolonged the life span of rats with cancerous liver and non-cancerous liver transplantation, highlighting its role in lowering the risk of recurrence and prolonging survival in cancer patients following liver transplantation (81).

## 4 S1P-S1PR is associated with liver injury in sepsis

### 4.1 Mechanisms associated with sepsis-induced liver injury

**Figure 1:**
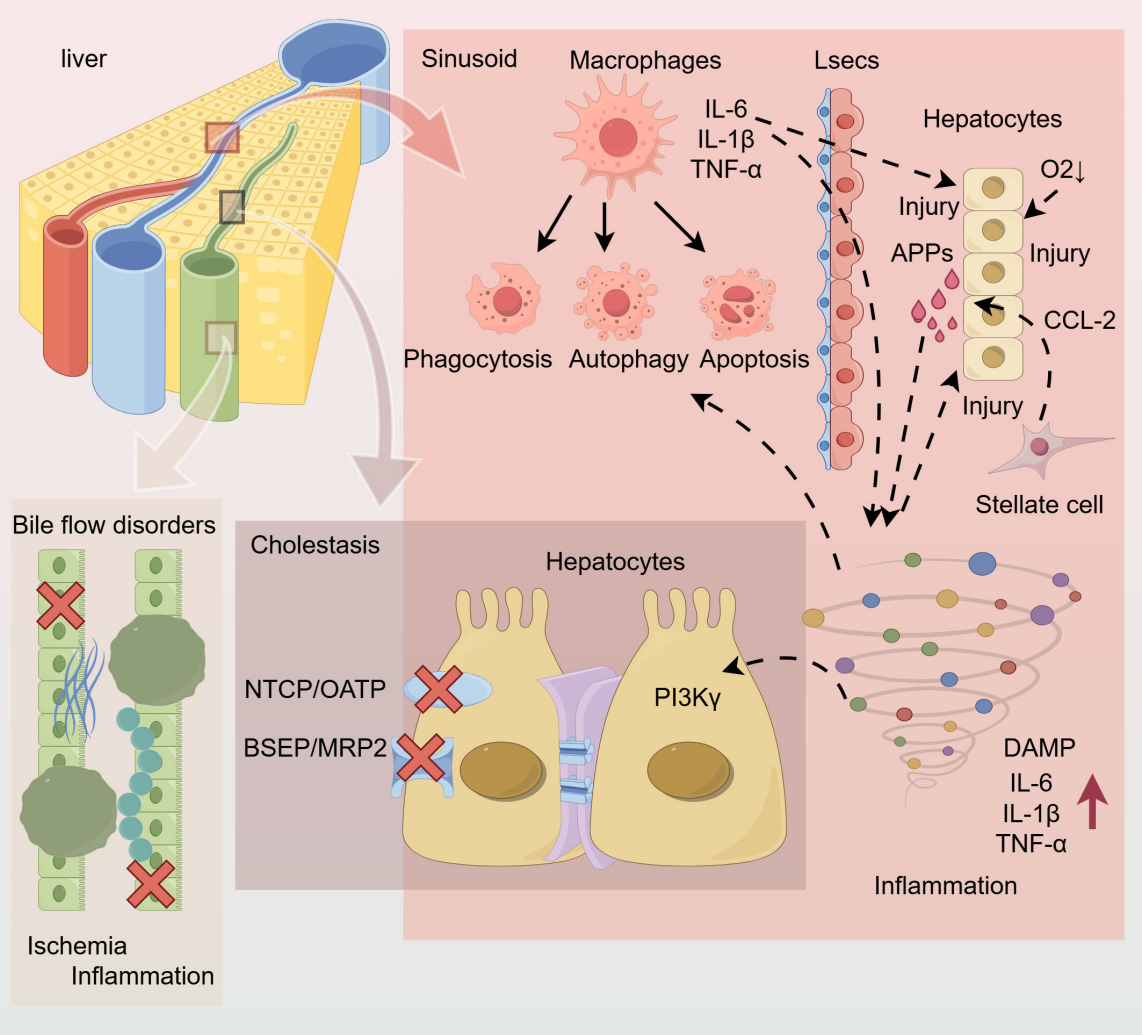
During sepsis, liver macrophages (Kupffer cells) in hepatic sinusoids synthesize large amounts of inflammatory cytokines (TNF-α, IL-6, IL-1β) through the TLR4-MyD88/NF-κB pathway. Moreover, stellate cells produce chemokine (CCL2), which attracts other immune cells and signals hepatocytes to synthesize acute-phase proteins APPs, amplifying the inflammatory effect and resulting in an inflammatory storm. When an inappropriate immune response or overwhelming inflammation occurs, high levels of DAMP and proinflammatory cytokines are generated in the liver, leading to significant hepatocyte damage, macrophage autophagy, and apoptosis. These cytokines also mediate damage to hepatocyte bile transport system (NTCP; OATP; BSEP; MRP2), which disrupts tight junctions and limits intracellular biotransformation, leading to bile metabolism disorders. In addition to impacting metabolism, inflammation and ischemia lead to bile duct structure damage, with bile flow defects further exacerbating cholestasis. TNF-α, tumour necrosis factor-alpha;IL-6, interleukin-6; IL-1β, interleukin 1 beta; TLR4, Toll-like receptor 4; MyD88,Myeloid Differentiation Primary Response Protein 88; NF-κB, nuclear factor κB; CCL2, C-C motif chemokine ligand 2; DAMP, damage-associated molecular pattern; NTCP, basolateral sodium taurocholate cotransporter; OATP, organic anion transporter; BSEP, tubular bile salt outlet pump; MRP2, tubular conjugate export pump.

Cholestasis is generally associated with sepsis (5). Early liver dysfunction (plasma bilirubin >2 mg/dL) was observed in 11% of critically ill patients in a large prospective multicenter study and was identified as a strong independent risk factor for late mortality (82). During sepsis, the liver is attacked by pathogens, toxins, or inflammatory mediators, and pathological changes can progress from hepatocyte dysfunction to liver injury, eventually culminating in liver failure (83). Liver dysfunction frequently occurs during the early phase of sepsis, primarily due to inflammation and hypoperfusion (84, 85). At the same time, inflammation also triggers the formation of ROS, leading to oxidative stress (86). Septic liver injury is characterized by the following clinical features: hypoxic hepatitis, sepsis-related cholestasis, and hyperbilirubinemia (7). On the one hand, persistent hepatic ischemia, hypoxia and inflammation cause oxygen metabolism within liver cells (87). On the other hand, energy deficiency, endotoxin, or proinflammatory cytokines may cause cholestatic hepatocyte injury by disrupting the outflow and excretion of bile (88). Besides, the hepatobiliary excretion mechanism is particularly sensitive to inflammation (**Figure 1**), which increases vulnerability to disruption in bile metabolism post-infection. A vicious cycle may be formed upon endotoxin-induced cholestasis, leading to a decrease in bile acid concentration in the small intestine, thereby driving bacterial translocation and endotoxin absorption and further aggravating cholestasis (89).

### 4.2 S1P-S1PR is associated with liver inflammation

Recent studies have concluded that S1P is associated with certain models of acute liver injury (90). Nevertheless, although S1P has been documented to contribute to liver injury in the majority of studies, it may exert protective effects in others. For instance, S1P participates in the recruitment and activation of immune cells during liver injury (71). However, *in vitro* studies have revealed that S1P protects hepatocytes from apoptotic injury (91).

Hyperactivation of the immune system is a common cause of acute organ injury, including the liver, wherein various liver-resident immune cells, such as Kupffer cells (KCS), liver natural killer T (NKT), and dendritic cells, play an instrumental role (92). This inflammatory cascade not only eliminates bacteria but also damages the liver (93). In an *in situ* perfusion experiment in piglets, endotoxins caused only pulmonary hypertension and neutropenia, while the levels of tumor necrosis factor α (TNF-α), interleukin (IL) -6, and nitric oxide (NO) were elevated when perfused into the lungs alone, without causing pulmonary edema (94). However, when the liver was included in the perfusion, the endotoxins caused marked hypoxemia and pulmonary edema with significant increases in the levels of TNF-α, IL-6, and NO (94). These findings conjointly suggest that the liver is a major source of cytokine and NO production following exposure to endotoxins (5).

S1P is a chief component in regulating the transport and activation of several immune cells (23). As an important immune organ in the body, the S1P signal transduction pathway is also involved in inflammatory liver injury (23). Indeed, in LPS/D-galactosamine (D-Gal)-induced liver failure, S1P levels were elevated, whilst the expression of SphK1, S1PR1, and S1PR3 was upregulated in the liver, and the levels of serum markers of liver failure, AST and ALT, and the serum cytokines TNF-α, IL-1, and IL-6 were increased (25). We speculate that SphK1 may play up-regulate the expression of S1P in peripheral blood monocytes, Kupffer cells, and liver resident macrophages (95). Treatment with the pan-SPHK inhibitor N, N-dimethylsphingosine reduced mortality in mice with sepsis-induced liver injury, as well as decreased liver inflammation and cell death (96). Furthermore, the Spns2/S1P signaling pathway has been shown to play a regulatory role at different stages of the inflammatory response. Specifically, the Spns2/S1P signaling pathway regulates the intensity of macrophage-mediated inflammation via the lactate-ROS axis during the early stages of sepsis (97). At later stages, it maintains the antimicrobial response of macrophages. Consequently, enhancing this pathway may restore the balance in immune responses during sepsis and prevent early hyperinflammation and late-stage immunosuppression (97). A previous study indicated that down-regulating the SPHK1/S1P/S1PR1 pathway can inhibit inflammation and oxidative stress, thereby relieving LPS-induced acute liver injury (25). Another study postulated that S1PR2 deficiency mediates the type 2 immune response and alleviates sepsis-induced lung injury by promoting IL-33 release from macrophages (98). Meanwhile, both S1PR2 gene deletion and pharmacological inhibition of S1PR2 significantly limited bacterial load, enhanced macrophage phagocytosis, and improved the survival rate of septic mice (99). It is worthwhile emphasizing that S1P promotes the activation of the NLRP3 inflammasome and contributes to the secretion of IL-1β and IL-18 in bone marrow-derived macrophages through S1PR2 (100). Conversely, S1PR2 knockdown inhibits the activation of the NLRP3 inflammasome in bone marrow-derived macrophages and alleviates liver inflammation (71, 100). Inhibition of S1PR3 can inhibit ATP-induced NLRP3 inflammasome activation in macrophages through TWIK2-mediated potassium efflux (101). Therefore, different S1PRs may operate together to optimize innate immunity and manage infection during sepsis.

In addition to macrophages, NK cells have been extensively studied. They constitute an important innate immune cell population in the liver, accounting for 30%-40% and 10%-20% of total intrahepatic lymphocytes in humans and mice, respectively (102). Importantly, NK cells play a critical role in the first-line innate defense and immune surveillance of the liver by eliminating invading pathogens, toxins, and circulating tumor cells. Moreover, they also mediate liver injury by interacting with cytokines such as IFN-γ and TNF-α (103). Mouse liver Kupffer cells are stimulated by bacterial superantigens to synthesize IL-12 and IL-18, which activate NK cells and NK1+ T cells to produce IFN-g (104). IL-12/LPS-activated NK1 T cells exhibited potent cytotoxicity against homologous liver cell lines *in vitro (* 105). Originating from the bone marrow (BM), NK cells are released into the circulation and are abundant in the spleen and liver (106). S1PR5 is a key chemokine receptor in NK cells that regulates the distribution of NK cells *in vivo* and their trafficking to lesion sites (107). In mice deficient in this receptor, NK cell distribution is altered, with decreased NK cell numbers in the blood and spleen and increased NK cell numbers in the lymph nodes (LN) and BM (108). Noteworthily, S1PR5 knockdown has been shown to reduce the number of circulating NK cells in the liver, spleen, and lungs of mice (109). S1PR5 is required for NK cell mobilization to inflammatory organs (108). Poly (I: C) is a compound that stimulates NK cells and induces the recruitment of NK cells from the spleen to the liver. After treatment with poly (I: C), wild-type NK cells accumulated in the liver (four to five-fold increase) and decreased in the spleen. At the same time, serum ALT/AST levels were marginally elevated, accompanied by mild inflammation and focal necrosis in the liver. In contrast, poly (I:C) treatment failed to cause significant changes in NK cell abundance in spleen or liver of S1PR5-deficient mice (110). PolyI: C induction also significantly up-regulated the expression of S1PR5 in the liver (111). Dong et al. demonstrated that the depletion of NK cells significantly attenuated PolyI: C-induced liver injury (112). Overall, these findings indicate that modulating the distribution of NK cells in the liver by regulating the expression of S1PR5 may be a new therapeutic approach for the treatment of sepsis-induced liver injury.

### 4.3 S1P-S1PR and biliary excretion function

Bile formation is an active osmotic process that involves the passive movement of organic and inorganic molecules across the tubular membrane and water and also necessitates the involvement of integrated membrane proteins, namely the intact cytoskeleton, tight junctions, and intracellular signaling (88). Bile acids are synthesized from cholesterol in hepatocytes and, after binding to glycine or taurine, are actively transported into the biliary system via ATP-binding cassette (ABC) transporters (113). Likewise, S1P can also be transported to the extracellular space by ATP-binding cassette (ABC) transporters (114). Hepatocytes secrete bile acids through bile salt export proteins (BSEP, ABCB11), ABCB4, and ABCG5/ABCG8 (115). An increasing number of *in vitro* studies have unveiled that LPS-induced proinflammatory mediators (including TNF-α) impair the downstream expression and functional integrity of hepatocyte transporters, such as BSEP and multidrug resistance-associated protein 2 (mrp-2) (116). Among them, the most prominent transport is the energy-dependent tubular ATP-binding cassette (ABC) transporter required for normal bile secretion, which underlies sepsis-associated cholestasis (117). This inflammatory shock may lead to permanent hepatic excretory dysfunction (85). ABCA1 and ABCG1 play a central role in cholesterol efflux from macrophages during atherosclerosis (118). In an earlier study, S1P and S1PR3 receptors were identified as modulators of ABCA1-mediated cholesterol efflux (118). The fact that S1PR3-deficient macrophages have lower ABCA1 gene expression and reduced cholesterol efflux suggests that sustained S1PR3 signaling is required to maintain basal cholesterol efflux (119). This finding led us to hypothesize the role of S1P and S1PR in the regulation of bile acid secretion in hepatocytes.

In sepsis, inflammatory factors may mediate sepsis-related excretory liver dysfunction through the PI3K pathway (85). Considering that bile acid-induced inflammasome activation has a synergistic effect with LPS, cholestasis further aggravates LPS-induced sepsis (120). It has also been found that in inflammatory cholangiopathies, proinflammatory cytokines stimulate biliary epithelium to produce NO through inducible nitric oxide synthase (NOS2) induction. In turn, NO causes ductal cholestasis through reactive nitrogen oxides (RNOS)-mediated inhibition of adenylate cyclase (AC) and cyclic adenosine monophosphate (cAMP)-dependent HCO3- and Cl- secretory mechanisms (116, 121). The use of JTE-013, a specific inhibitor of S1PR2, may improve cholestatic liver injury induced by common bile duct ligation by reducing hepatitis response and apoptosis (122). Nonetheless, the mechanism by which JTE-013 ameliorates liver injury remains elusive, and its relationship with the PI3K pathway and NO warrants further exploration.

### 4.4 S1P-S1PR and the liver-intestinal circulation

Ascribed to anatomical connections, the theory of hepato-enteric circulation is widely accepted, whereby the gut and liver communicate bi-directly through the biliary tract, portal vein, and systemic circulation (123). At present, existing evidence suggests that the enterohepatic crosstalk between the gut and the liver through bacterial translocation and liver-derived molecules, such as bile acids, play a crucial role in the pathogenesis of sepsis (123). Due to bacterial invasion during sepsis, intestinal barrier disruption and intestinal microbiota dysregulation result in the translocation of intestinal pathogen-associated molecular patterns (PAMP) and damage-associated molecular patterns (DAMP) to the liver and systemic circulation (9). Under physiological conditions, the liver regulates immune defense through mechanisms such as bacterial clearance, cytokine production, acute-phase protein release, and regulation of inflammatory metabolism (124). However, during systemic infections, these inflammatory responses may become excessive, leading to impaired pathogen clearance and impaired hepatic metabolism, which can lead to further damage to the gut barrier (89).

The gut barrier consists of a single-cell layer of epithelium, the local immune system, and the microbiome (125). This barrier includes a mechanical component, namely the biological structure composed of intestinal epithelial cells (IEC) and vascular endothelial cells (EC) (126). Various tight junctions (TJS) are present between cells, which comprise various tight junction proteins and cadherins, such as including ZO-1, occludin, cadherin, claudin, and β-catenin. These junctions can resist the invasion of pathogens and harmful substances from infiltrating the intestinal mucosa and prevent the exudation of substances in the intercellular space (127). From the pathophysiology of intestinal injury, cellular alterations and bacterial toxins can induce intestinal edema and intestinal dysfunction by disrupting normal intercellular junctions, reducing vascular endothelial cadherins and TJS, and increasing vascular permeability (128). In preclinical models of sepsis, changes in tight junctions were observed after 1 hour of sepsis onset, with intestinal hyperpermeability persisting for at least 48 hours (125). LPS, a component of the outer wall of Gram-negative bacteria, alters TJ protein assembly, leading to small intestinal leakage, and regulates the inflammatory response through TLR4, further aggravating the changes in TJ (129).

**Figure 2:**
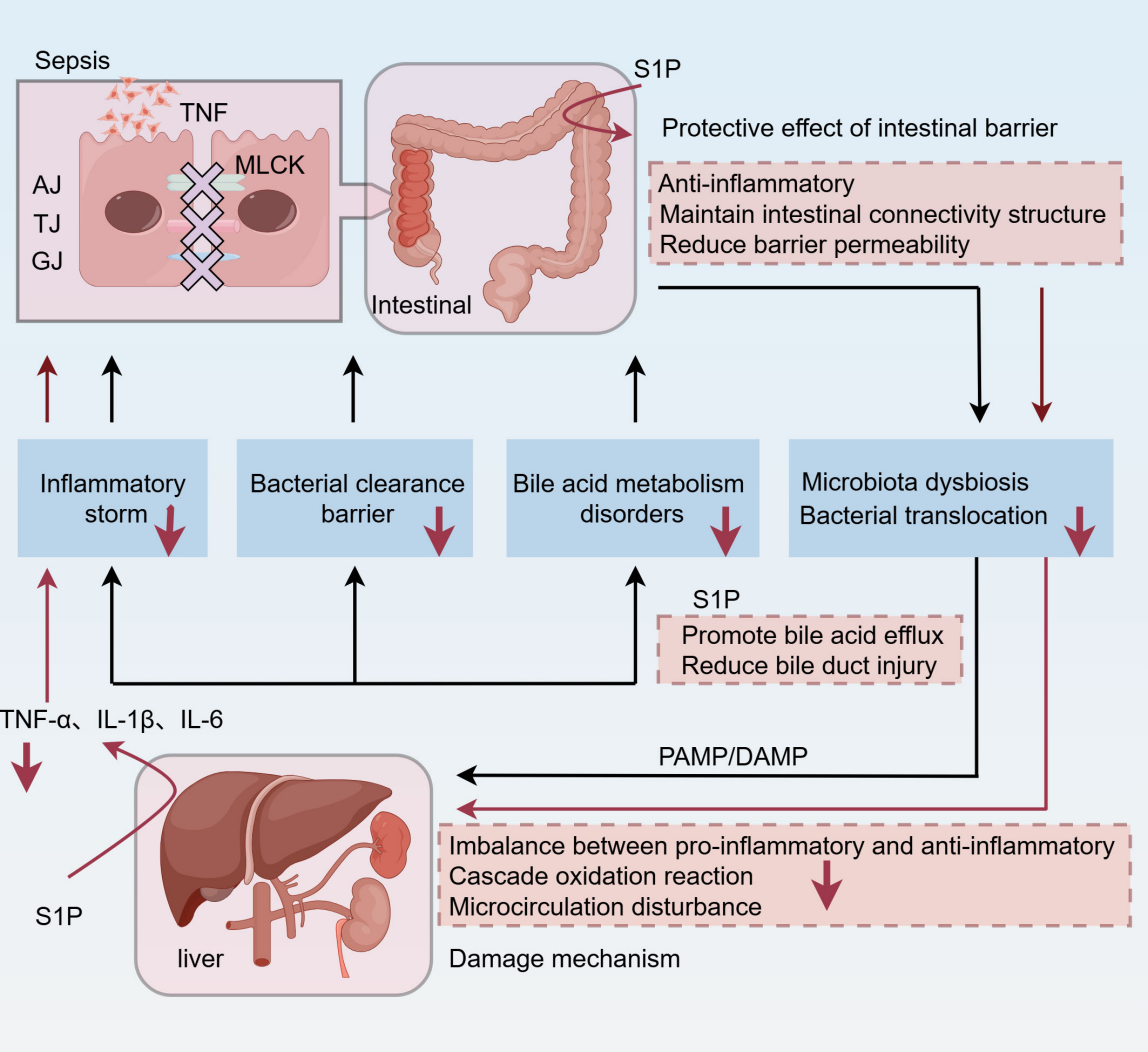
During sepsis, bacterial inflammation leads to the destruction of intercellular junctions in the intestinal barrier, including AJ, TJ, and GJ. In addition, MLCK is associated with inflammatory activation. As a result, the gut’s primitive microbial flora is dysregulated, and bacteria are translocated into the blood. Two injury patterns, PAMP and DAMP, reach the liver via the portal vein and hepatic artery. As the first line of defense, the liver is also damaged by the imbalance between pro-inflammatory and anti-inflammatory factors, the peroxidation cascade, and microcirculation disorders, resulting in inflammatory storms, bile acid metabolism disorders, and impaired bacterial clearance. Among them, elevated bile acid levels exacerbate inflammation and oxidative stress and lead to hepatocyte apoptosis or necrosis. Interestingly, the gut-liver axis further amplifies these effects. Increased intestinal permeability leads to increased systemic inflammation through a positive feedback loop. S1P can enhance liver function and concurrently alleviate liver injury by protecting intestinal barrier function, inhibiting inflammation, minimizing bacterial translocation, and improving bile excretion. TNF, tumor necrosis factor; AJ, adherens junctions; TJ, tight junctions; GJ, gap junctions; MLCK, myosin light chain kinase; PAMP, pathogen-associated molecular pattern; DAMP, damage-associated molecular pattern.

S1P has been described as a barrier-enhancing molecule (130). In a clinical study, plasma S1P levels were reduced, and VE-cadherin levels were increased in patients with septic shock (22). S1P can up-regulate the expression of cell junction-related proteins such as E-cadherin and ZO-1 and promote VE-cadherin translocation, thereby strengthening the intestinal epithelial barrier and reducing the permeability of the intestinal epithelial layer, reducing sepsis-related intestinal injury, and improving the survival rate in sepsis (131, 132). In addition, a significantly negative correlation was noted between the severity of endothelial dysfunction and plasma S1P concentration in sepsis (133). Recent studies have hypothesized that S1P regulates EC biology via various molecular mechanisms, including vascular development and angiogenesis, inflammation, permeability, and the production of reactive oxygen species (ROS), nitric oxide (NO), and hydrogen sulfide (H_2_S) (134). During sepsis, S1P interacts with the receptors S1PR1 on EC to reduce vascular permeability and maintain the EC barrier through the aforementioned mechanisms (135). Therefore, we posit that S1P plays a major role in intestinal barrier injury during sepsis. In an earlier study investigating alcoholic liver disease, treatment with citrate-ethanol-derived red rice seed skin extract (RRA) increased SPHK2 and S1P levels, improved gut microbiota composition, restored the integrity of the intestinal barrier, reduced plasma and liver LPS levels, and inhibited the activation of the Toll-like receptor 4 (TLR4)/nuclear factor κB (NF-κB) pathway, as well as alleviated liver pathological damage and oxidative stress, reduced inflammation and apoptosis, and enhanced liver function (136). Consequently, alleviating damage to the intestinal barrier function may attenuate hepatic injury (**Figure 2**). However, not all S1P receptors contribute to barrier enhancement (137). For example, intravascular administration of S1P can prevent inflammatory damage associated with acute lung injury. Conversely, elevated S1P concentrations *in vitro* may lead to severe disruption of the endothelial cell barrier, potentially through engagement of S1PRs other than S1PR1 (137).

## 5 Application of S1P and its receptor pathway-related drugs in sepsis-induced liver injury Resource Identification Initiative

S1P plays a significant role in inflammatory responses, immune regulation, and the maintenance of the intestinal mucosal barrier, and is considered a potential therapeutic biomarker for sepsis (134). In the S1P transduction pathway, some drugs can alter the physiological function of S1P in various diseases by targeting S1P receptors. In addition, sphingosine kinase (SPHK) and S1P lyase (S1PL) are key enzymes that affect the physiological functions of S1P. Increasing or decreasing the activity of these corresponding enzymes to regulate the concentration of S1P in different cells represents a promising treatment for diseases (138).

Fingolimod (FTY-720) is an oral immunomodulatory drug (18) that can competitively bind to S1PR1, leading to receptor internalization and inhibiting the egression of lymphocytes from secondary lymphoid organs, thereby exerting immunosuppressive effects (139). After oral administration, the plasma concentration of FTY720 gradually rises to its peak approximately 12 to 24 hours post-dose (140). Recent research suggests that it is also effective for the treatment of ulcerative colitis (141). In a previous study, FTY-720 reduced S1PR1 levels, inhibited the NF-κb/IL-6/STAT3 cascade, and delayed the progression of colitis-related tumors (142). Furthermore, FTY-720 ameliorates diabetes-induced liver injury by inhibiting oxidative stress and inflammation (143). In preclinical animal models of hepatopulmonary syndrome, FTY-720 reduced systemic inflammation, portal pressure and liver fibrosis, significantly improving the survival rate of the animals (144). Additionally, preliminary studies suggest that FTY-720 may have potential antibacterial effects (145). The above-mentioned results signal that FTY-720 may modulate the regulation of the NF-κB pathway, and its use in sepsis-induced liver injury may inhibit inflammation and oxidative stress. Therefore, FTY-720, as an S1P agonist, holds promise as a candidate hepatoprotective agent.

With the deepening of clinical research, it was found that FTY-720 has low receptor selectivity (146). While activating S1PR1, it also activates S1PR3, leading to the activation of G protein-gated inward rectifier potassium channels in the myocardium, thereby causing adverse reactions such as bradycardia and atrioventricular block. In addition, other side effects have been reported, including headache, flu-like symptoms, diarrhea, back pain and cough, with an incidence rate of 10% or higher; other less common adverse events include elevated liver enzyme levels (8%), macular edema (0.4%) and hypertension (6.5%), which greatly limit its application (147). However, clinical studies have shown that the concurrent use of silymarin can reduce liver complications caused by FTY-720 (148). The effects of FTY-720 on multiple S1P receptor subtypes explain its known and some unexpected adverse reactions. Moreover, new-generation drugs have also been proposed. Siponimod (BAF312) is a dual agonist of S1PR1 and S1PR5, with a selectivity for S1PR1 and S1PR5 about 1000 times higher than that for S1PR2-4, and can significantly and persistently induce the internalization of S1PR1 (18). Current data show that BAF312 has been approved for use in patients with active secondary progressive multiple sclerosis (149). Due to the weak activation of S1PR3 by BAF312, cardiac adverse reactions are less (150). However, other adverse reactions still exist, such as hypertension, lymphopenia, cystoid macular edema and convulsions (150).

Alternatively, Ozamomod is a sphingosine-1-phosphate (S1P) receptor modulator with high affinity for S1P isoforms 1 and 5 (S1P1 and S1P5) (151). In a phase III trial, Ozanimod outperformed placebo as an induction and maintenance therapy in patients with moderately to severely active ulcerative colitis (152). Currently, Ozamomod is approved by the US Food and Drug Administration (FDA) and the European Medicines Agency for the treatment of UC. Notably, S1PR1 agonists can improve barrier function by restoring TJ proteins and inhibiting IEC cell apoptosis (153). Moreover, it can inhibit bacterial translocation by alleviating intestinal barrier damage during sepsis and the liver immune response. Nonetheless, its effects on S1PR5 and in reducing the number of NK cells in the liver to alleviate inflammation remain elusive.

## 6 Conclusions and views

S1P and its receptors play key roles in the immune and metabolic processes of the body and are closely related to various inflammatory responses, oxidative stress, and lipid deposition diseases. As an integral organ for immunity and metabolism, the liver not only protects against the primary attack of bacterial toxins during sepsis but also regulates the metabolism of deposited substances after the attack. S1P and S1PR (1–3) are highly expressed in the liver and can act through various signaling pathways. For instance, the Spns2/S1P signaling pathway plays an inhibitory role during the early stage of the inflammatory response to prevent inflammatory storms and plays a promoting role at later stages to reduce damage caused by immune suppression. Additionally, S1P likely plays an essential role in the regulation of bile acid metabolism, which is particularly relevant in the context of liver injury. Further research on the mechanism of mutual regulation of liver injury, immunity, and metabolism may assist in the discovery of novel therapeutic targets. At present, S1PR drugs and other modulators have shown efficacy in regulating immunity. Thus, future studies should explore new metabolic targets to advance treatment strategies for inflammatory and metabolic disturbances linked to sepsis-induced liver injury.

To date, S1P production has mainly relied on chemical synthesis or blood extraction, but these methods suffer from the limitations of complexity and high cost. To address this problem, a novel approach has been proposed: S1P production using Saccharomyces cerevisiae strains (154). This not only provides new ideas for the genetic manipulation of sphingolipid derivatives, but also demonstrates the potential of biosynthesis in this field. Accurate quantification of S1P, a membrane phospholipid metabolite, has been a challenge. To this end, liquid chromatography-tandem mass spectrometry (LC-MS/MS) was introduced to enable a comprehensive analysis of these lipids and can be used for S1P analysis in plasma and extracellular vesicle components such as macrophages-derived vesicles (155). Furthermore, we observed that S1P concentration changes in blood are different from those in organ tissues after the onset of sepsis. This difference may be due to changes in the total amount or redistribution of S1P *in vivo*, but the specific regulatory mechanism remains unclear. Future studies are needed to investigate whether S1P regulation of different organs is influenced by the S1PR expression in the organ itself, especially those S1PR that are difficult to measure with existing techniques (such as S1PR4/5 in the liver). These receptors may play an important role through pathway amplification effects rather than solely dependent on their own expression levels. Finally, the determination of S1P levels in bile and its target organs, such as liver, bile ducts, and intestine, under physiological conditions is essential for a deeper understanding of the pathological changes of S1P in hepatobiliary and digestive diseases. It should be noted that there are differences in animal and human metabolic levels and therefore should be taken into account in studies. As more and more scholars focus on the role of S1P, it is believed that these problems will be solved in the future.
